# Safe Birth and Cultural Safety in southern Mexico: study protocol for a randomised controlled trial

**DOI:** 10.1186/s13063-018-2712-6

**Published:** 2018-07-04

**Authors:** Iván Sarmiento, Sergio Paredes-Solís, Neil Andersson, Anne Cockcroft

**Affiliations:** 1 CIET/PRAM, Department of Family Medicine, McGill University, 5858 Chemin de la Côte-des-Neiges 3rd Floor, Suite 300, Montreal, H3S 1Z1 Quebec Canada; 2 Escuela de Medicina y Ciencias de la Salud, Universidad del Rosario, Bogotá, Colombia; 3 Centro de Investigación de Enfermedades Tropicales (CIET), Universidad Autónoma de Guerrero, Calle Pino s/n Colonia El Roble, postal code 39640 Acapulco, Guerrero Mexico

**Keywords:** Traditional midwives, Cultural safety, Epidemiology, Randomised Controlled Trial, Equity in access, Aboriginal health

## Abstract

**Background:**

Indigenous women in the southern Mexican state of Guerrero face poor maternal health outcomes. Living as they do at the very periphery of the Western health system, they often receive low-quality care from health services that lack human and financial resources. Traditional health systems remain active in indigenous communities where traditional midwives accompany women through motherhood. Several interventions have explored training birth attendants in Western birthing skills, but little research has focussed on supporting traditional midwives by recognising their knowledge. This trial supports traditional midwifery in four indigenous groups and measures its impact on maternal health outcomes.

**Methods:**

The study includes four indigenous populations in the State of Guerrero (*Nahua*, *Na savi*/Mixteco, *Me’phaa*/Tlapaneco and *Nancue ñomndaa*/Amuzgo), covering approximately 8000 households. A parallel-group cluster-randomised controlled trial will compare communities receiving usual care with communities where traditional midwives received support in addition to the usual care. The intervention was defined in collaboration with participants in a 2012 pilot study. Supported midwives will receive a small stipend, a scholarship to train one apprentice, and support from an intercultural broker to deal with Western health personnel; additionally, the health staff in the intervention municipalities will participate in workshops to improve understanding and attitudes towards authentic traditional midwives. A baseline and a final survey will measure changes in birth and pregnancy complications (primary outcomes), and changes in gender violence, access to healthcare, and engagement with traditional cultural activities (secondary outcomes). The project has ethical approval from the participating communities and the Universidad Autónoma de Guerrero.

**Discussion:**

Indigenous women at the periphery of Western health services do not benefit fully from the attenuated services which erode their own healthcare traditions. Western health service providers in indigenous communities often ignore traditional knowledge and resources, inadvertently or in ignorance, disrespecting indigenous cultures. Improved understanding between midwives and the official healthcare system can contribute to more appropriate referral of high-risk cases, improving the use of scarce resources while lowering costs of healthcare for indigenous families.

**Trial registration:**

ISRCTN12397283. Retrospectively registered on 6 December 2016.

**Electronic supplementary material:**

The online version of this article (10.1186/s13063-018-2712-6) contains supplementary material, which is available to authorized users.

## Background

Maternal mortality and morbidity remain inequitable burdens for indigenous women in Mexico, as in many other countries [1, 2]. Modern obstetric care, especially in emergencies, can be key to survival [3, 4] and, in remote indigenous communities where the needs might be more pressing, women almost invariably receive poorer-than-average health services [5, 6]. Inappropriate allocation of state resources and weakness of local governments are part of the problem on the supply side [7]. On the demand side, lack of interaction with traditional knowledge systems in Western medical facilities has led many indigenous women to shun Western health services [8]. One consequence, in many remote indigenous communities, is that traditional midwives are the only source of care available for maternal health [9].

Much of the research to address this state of affairs focusses on short-term training of non-traditional task-oriented birth attendants, and training of traditional midwives in Western birthing concepts and practices [9–12]. A systematic review summarising 60 experimental and quasi-experimental studies of training traditional birth attendants (TBAs) found a small reduction of perinatal and postnatal mortality, and that trainees remembered the content of their training (‘improvement in knowledge’) [13]. A 2011 meta-analysis synthesised six cluster-randomised controlled trials (RCTs) of *training and support* of TBAs [14]. All six RCTs found a reduction in perinatal death (Number Needed to Treat (NNT) 35, 95%CI 24–70) and neonatal death (NNT 98, 95%CI 66–170). Three of the RCTs reported on maternal mortality and showed a non-significant reduction.

A 2009 systematic review found ‘low/moderate-quality evidence’ suggesting that training TBAs ‘may improve linkages with facilities and improve perinatal outcomes’, and meta-analysis showed an 11% reduction in intrapartum and intrapartum-related neonatal mortality [12]. A synthesis of systematic reviews published in 2014 concluded that in low- and middle-income countries training TBAs, ‘as a part of community-based intervention packages showed significant improvement in referrals (RR 1.4, 95%CI 1.19–1.65)’, ‘significant reductions in maternal morbidity (RR 0.75, 95%CI 0.61–0.92), neonatal mortality (RR 0.76, 95%CI 0.68–0.84) and perinatal mortality (RR 0.80, 95%CI 0.71–0.91)’ [15]. The success of programmes was found to be context specific [16], and related to better communication with formal healthcare systems [16–18].

Throughout the academic literature, the term ‘birth attendant’ instead of ‘midwife’ ignores cultural issues and the experience and full social role of traditional midwives [19]. The research focus on training assumes the inferiority of traditional midwifery, or their lack of competence in birthing techniques [20]. The emphasis is on compliance with Western midwifery, rather than on the strengths of traditional midwifery [10]. The World Health Organisation (WHO) excludes traditional midwives from the category of skilled birth attendants, reserving this term for those midwives with formal Western training [7, 9]. We have not found any published RCT that tests the value of *supporting* the original practices of traditional midwives.

### Terminology: authentic traditional midwives

Birth traditions in most indigenous cultures involve the support of a traditional practitioner, frequently called in the academic literature untrained traditional birth attendants (TBAs) [9, 21]. To clarify terminology, we distinguish between (1) *authentic traditional midwives,* whose recognition by their communities is reflected in the number of births they attend each year and the traditional knowledge they hold, (2) *casual* or *coincidental birth helpers*, who might help in a family or neighbourhood emergency and (3) *skilled* or *trained birth attendants*, often conflated by acronym with TBAs, who attend courses in Western birth practices and who might receive official certification.

Our concern in this trial is exclusively authentic traditional midwives, recognised in their own cultures and accessed by their communities. We prefer not to abbreviate the term, in order to avoid confusion with Western concepts of trained birth attendant or TBA. For economy of words we refer to them as traditional midwives.

Traditional midwives are part of the traditional health system of their communities [22]. Beyond their technical role in pregnancy and birth, traditional midwives are counsellors and indigenous knowledge bearers, transmitters of culture and cultural values [23]. Some traditional midwives take government training courses, similarly to the ‘skilled’ or trained birth attendants, when these courses allow traditional midwives to obtain birth certificates for the children they deliver. Some traditional midwives might incorporate aspects of Western obstetrics; for example, cutting of the umbilical cord, into their practice [24]. What distinguishes traditional midwives is their rootedness in community and culture, and this is confirmed by the confidence placed in them by their communities. Usually female – the *Me’phaa* or Tlapaneco of Guerrero also have male *parteros –* they accompany the pregnancy, attend the birth and advise on care of the newborn [25, 26].

### The pilot study

A pilot cluster-RCT tested the feasibility and acceptability of an intervention to support authentic traditional midwives between 2008 and 2012 [27]. The pilot was not powered to determine the effect of the intervention, but it did measure outcomes in the intervention and control group, in order to establish that the intervention was not likely to have an adverse effect on maternal morbidity and mortality.

The pilot study was conducted in Xochistlahuaca municipality with *Nancue ñomndaa* (Amuzgo) communities and included 16 indigenous women clearly considered to be traditional midwives by the communities. These traditional midwives were randomly assigned into two groups, one of which received a co-designed intervention [28].

Each intervention midwife received financial support to pay an apprentice (about US$8 per month); had access to a local birthing centre (purpose-built, rented or loaned); and received logistical support from a male community health worker who could arrange transport for women referred to the local hospital and who could interact with the hospital staff on behalf of the traditional midwives, many of whom could not speak Spanish. Control communities continued receiving usual care, provided mainly by the healthcare centre (*hospital básico comunitario*) located in the municipal capital of Xochistlahuaca and by traditional midwives without external support. An unknown proportion of indigenous women in the rural areas of the municipality did not receive healthcare either from Western health staff or from traditional midwives.

The pilot showed that a larger trial would be feasible. It allowed us to adjust the intervention, to design and test questionnaires, to establish the local capacity needed to conduct a larger study, and to identify costs of the intervention. The pilot established the acceptability of the intervention according to three criteria. First, the intervention was safe; the groups with midwives receiving support did not have worse health outcomes and did not report complicated cases related to the intervention (see below). Second, the communities did not react against the recovery of traditions; some previous experience had suggested that some community members, particularly the younger ones, might interpret an intervention to support traditional midwives as an attempt to reduce the services provided by the Government. Third, the staff at the local healthcare centres accepted an increased involvement of midwives with no conflicts which would make the health authorities stop the intervention.

The pilot found similar levels of *pregnancy* complications between women in exposed communities (24/94) and controls (65/252) (OR 0.99, 95%CI 0.52–1.71). It was not intended to measure mortality but, in the event, results were compatible with a positive effect of supporting traditional midwives on reducing *birth* complications (9/91 exposed and 57/248 controls reported birth complications, OR 0.37, 95%CI 0.11–0.73). Women living in the intervention area did not report any neonatal deaths during the last year of the intervention (0/93, compared with 6/254 in control area, chi-square = 2.2, *p* = 0.13). The pilot also suggested advantages for women in terms of skilled birth attendance (92/94 among exposed and 233/253 among controls were assisted by a traditional midwife or physician, OR 3.95, 95%CI 1.0–15.59).

The significantly lower birth complications in intervention communities were likely due to two factors: (1) improved referrals as a result of the intercultural brokerage; and (2) increased use of traditional midwives in the intervention area, resulting in fewer women giving birth without a skilled birth attendant. The pilot demonstrated acceptability of the intervention among the communities and the economical and logistical feasibility of supporting traditional midwifery. The pilot also built local capacity for intercultural and multi-disciplinary research that is scientifically valid and also takes full account of the local cultural context.

### Objectives

The overall objective is to reduce maternal morbidity and mortality in indigenous communities without further marginalising or undermining their cultures. The overall hypothesis is that recovery and strengthening of traditional healthcare have a positive impact on indigenous people’s health. An explicit intention is to develop an intercultural approach that reduces the dependence on external resources and promotes the cultural assets of indigenous communities.

Specific objectives of the study are: (1) to assess the impact on maternal health outcomes of a co-designed intervention to support traditional midwives in four municipalities of Guerrero; (2) to assess the secondary or social outcomes of this intervention, including gender violence against pregnant women and behaviours related to traditional midwives; and (3) to evaluate the economic cost of the intervention.

Research question: Among the four main indigenous groups in Guerrero, does support for authentic traditional midwives lead to non-inferior maternal health outcomes and improved social outcomes within the study period, when compared with usual care?

Theory of change: Intercultural brokers increase effective contact with Western health services; this improved referral generates better maternal outcomes by allowing obstetric attention to focus on those who need it most. Better maternal outcomes, along with the apprentices and economic support provided by the intervention, increase prestige of traditional midwives within the communities. Midwives’ prestige promotes cultural continuity and strengthens the social fabric. Additionally, this prestige expands their services among women who do not need specialist obstetric intervention, thus decreasing pressure on poorly funded healthcare services. The no-longer-overloaded healthcare services are then better able to deal with emergency cases and those in need of Western obstetric care, which further improves maternal outcomes.

## Methods

### Design of the study

**Fig. 1:**
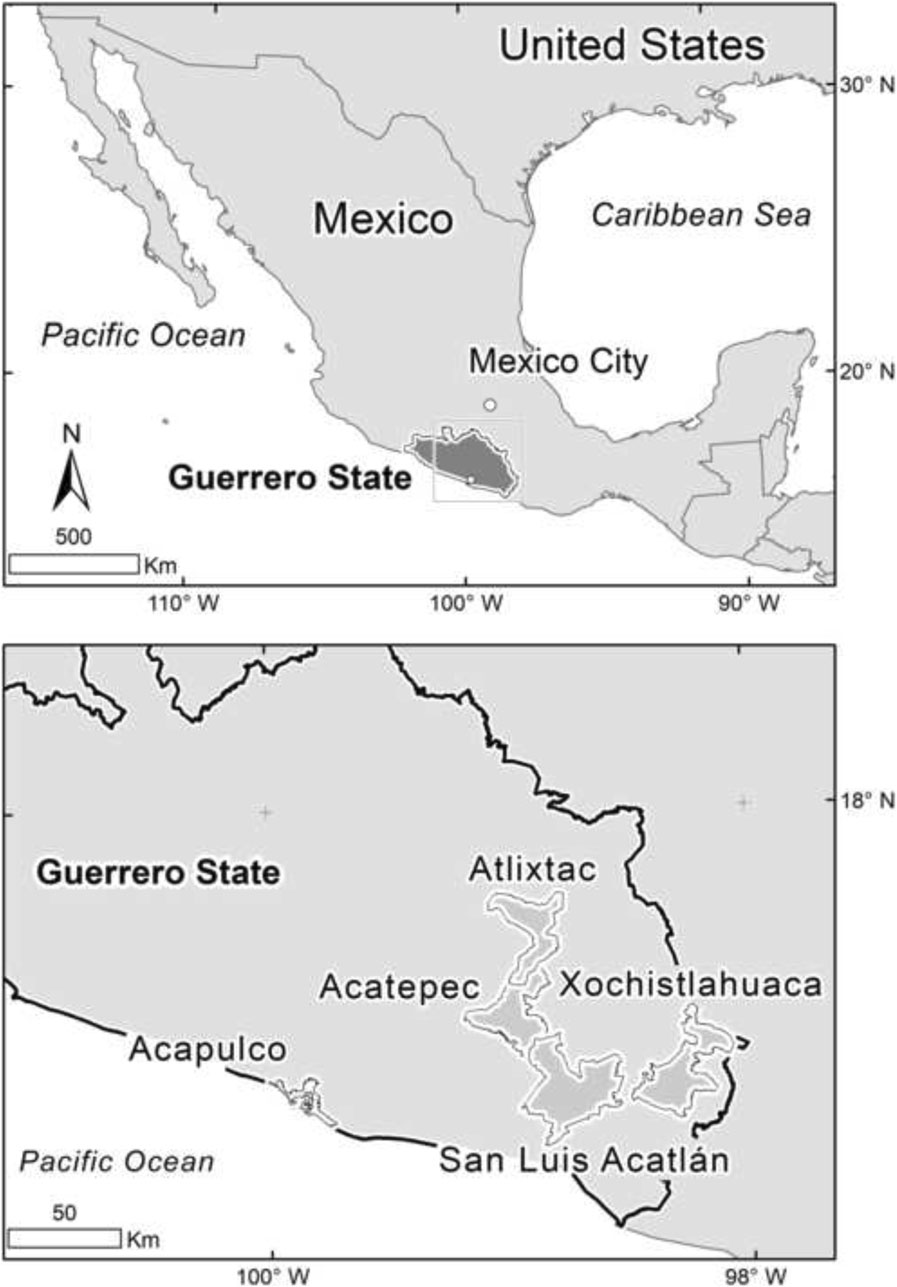
Map of the participating municipalities

A parallel-group pragmatic cluster-RCT will test the non-inferiority of maternal health outcomes of an intervention to support authentic traditional midwives in four indigenous groups (*Me’phaa, Nahua, Na savi* and *Nancue ñomndaa)* in four municipalities (Atlixtac, San Luis Acatlán, Acatepec and Xochistlahuaca) in Guerrero State (Fig. 1) [28].

### The setting

Indigenous people make up one third of the world’s poorest rural people, and this is also true of indigenous people living in Guerrero, currently Mexico’s third poorest state [29, 30]. Of the 481,000 indigenous people in the state, *Nahua* make up 40%, *Na savi* (Mixteco) 28%, *Me’phaa* (Tlapaneco) 22% and *Nancue ñomndaa* (Amuzgo) about 9%. They live in scattered and often remote communities with poor access to government services and rely mainly on subsistence agriculture. Most speak their traditional languages and self-identify as indigenous. Government-conditional cash-transfer programmes give a monthly US$15 incentive to indigenous women for improving attendance to official healthcare services and food consumption. Indigenous people in Mexico have less than average access to the country’s main health insurance system, and indigenous peoples of Guerrero state have the lowest access among indigenous groups nationally [31].

Where they are available outside of the cities, health services are often poorly staffed and of poor quality. In part, this is due to lack of qualified medical personnel. In the Montaña region of Guerrero, home to the *Na savi* and *Me’phaa* peoples, there are no obstetric services within 1 days’ travel for the population of several hundred thousand. Only one in four of Mexico’s indigenous women has completed secondary education, a requirement for training as a ‘skilled birth attendant’ in government programmes.

In Mexico, as elsewhere in Latin America, maternal and perinatal mortality among indigenous peoples is poorly documented. Indicators of indigenous maternal and child health in Guerrero State are below the national average, and maternal deaths are three times more common than in the non-indigenous population [2, 32]. Maternal mortality is five to six times the national average (281/100,000 in Zona Centro de Guerrero, compared with 51 in Mexico at large) and infant mortality three to four times higher (89 compared with 28 per 1000) [32, 33].

### Participants

Eighty indigenous communities in four municipalities with a total of around 8000 households. The study will include all indigenous women who give birth or become pregnant during the study period, and their adult family members.

### The intervention

The intervention has four components that incorporate the co-design exercise from the pilot study and subsequent discussions with the midwives in the four indigenous groups. The intervention comprises activities to invigorate the practice of traditional midwifery and increase the interaction of traditional midwives with the Western healthcare system. The intervention does not define a protocol for the management of motherhood in these communities; thus, Western physicians and traditional midwives remain autonomous in their own practice.

Component 1. Material support for 30 authentic traditional midwives. Each traditional midwife in the intervention group will receive a monthly stipend of US$20. This small financial support is meant to allow the traditional midwives access to basic goods and increase the time that they have available for their practice and patient care; most of these traditional practitioners are low-income elderly depending on their own work or on support from their families. Additionally, the small monthly payment will be a symbol of external esteem for the role of these traditional midwives, thus increasing their recognition among community members. Field coordinators will be in charge of the payments to the traditional midwives in the intervention municipalities.

Component 2. Scholarship support of one apprentice for each midwife. The midwives in the intervention group will each appoint one apprentice to receive a monthly stipend of US$10; the midwife will decide on the training programme and the criteria to evaluate the achievements of her apprentice. The midwife will authorise the payment for the apprentice, while the field coordinators will be in charge of the disbursement. The apprentices will support the practice of the traditional midwives, particularly in tasks that the midwives can no longer perform due to their age. This component will foster the intergenerational transfer of traditional midwifery practice and increase its recognition by community members.

Component 3. Improving understanding and attitudes of staff in the local government health centres towards traditional midwives. In this component, senior researchers from the *Centro de Investigación de Enfermedades Tropicales* in the *Universidad Autónoma de Guerrero* (CIET) will lead a workshop in each municipality to present evidence about the role of traditional midwives and the importance of intercultural skills for Western medical practice. The workshop participants will be the personnel from two primary healthcare centres and ten rural health posts in the intervention municipalities. The workshops will focus on presenting technical data to the staff and will not include traditional midwives, to avoid potential confrontation during this initial stage. Although we expect changes in the attitudes of the staff in the intervention municipalities, their clinical practice remains independent of the project.

Component 4. Training of intercultural brokers (*técnicos interculturales de salud*). A total of 17 community-appointed people will receive training. Inclusion criteria are: being a member of the relevant ethnic group and having basic understanding of traditional culture and Western health services. Each community will follow their own customs to select the candidates.

The training programme will build on previous experiences from Colombia tailored to local conditions of Guerrero [34], and its content will be organised into three thematic lines: culture, nature and health (Table 1). This triple thematic approach reflects a concept of health promotion that seeks to implement actions with positive impact not only on individual health but also on the cultural and environmental domains. Each thematic line comprises theoretical and practical sessions totalling 280 hours of class in 2 months. The training will take place in Acapulco, under the supervision of CIET and with support from Colombian instructors from the Centre for Intercultural Medical Studies. The project will provide accommodation and food for the trainees in Acapulco.

**Table 1:** Content of the course for training intercultural brokers in Guerrero State (May to June 2015)

Content	Thematic line
*Introductory module*	
Western medicine, biomedical model and traditional health	Health
Memory, will, and concepts about medicinal plants	
Traditional concept of heat and cold	
Self-care	
Nature and environment	Nature
Culture and intercultural dialogue	Culture
Traditional knowledge	
*Module of applied concepts*	
Cultural context and identity in Mexico	Culture
National and international legislation on behalf of indigenous peoples	
Internet, accounting basics and management	
Cultural diversity	
Oral tradition	
Traditional values and principles	
Indigenous education	
Basics of ecology	Nature
Soils and organic fertiliser	
Participatory mapping	
Tools for nature observation	
Biological diversity and its relation with cultural diversity	
Territories conserved by indigenous communities	
Food sovereignty and local food	
The health system of Mexico and official health programmes	Health
The human body	
Vital signs	
Nutrition	
First aid and injections	
Management of emergencies	
Wound care	
Most prevalent health problems in Guerrero (dengue, chikungunya, skin disorders, scorpion sting, diabetes, violence and oral health)	
Healthcare of a healthy child	
Healthcare of a sick child (undernourishment, acute diarrhoea, acute respiratory infection, intestinal parasitic infections)	
*Final cross-cutting module*	
Women’s health	
Self-care promotion	
Support of traditional midwifery	
*Practices and fieldwork*	
Practice: building a planting bed	
Fieldwork: nature observation and planting bed	
Fieldwork: botanical garden	
Fieldwork: archaeological sites	

Another guiding principle of the training programme for the intercultural brokers is the promotion of intercultural dialogue between indigenous and Western cultures [35]. This principle is the basis for the intercultural brokerage that the trainees will undertake when they return to their communities [36].

Once in their communities, the brokers will design a work plan applying the course contents to the specific needs that they identify for their communities. Each broker will support one to two midwives, and together they will cover two to three contiguous enumeration areas. The brokers will define these plans in consultation with the traditional midwives supported by them. The plan will consist of two linked components: activities to accompany the traditional midwives and actions for health promotion with an emphasis on women’s and maternal health. These activities will follow a pattern of implementation where the brokers will start with activities applying the contents learned during the training upon themselves, then they will involve their families and, finally, with increasing confidence, they will involve other members of their communities.

**Fig. 2:**
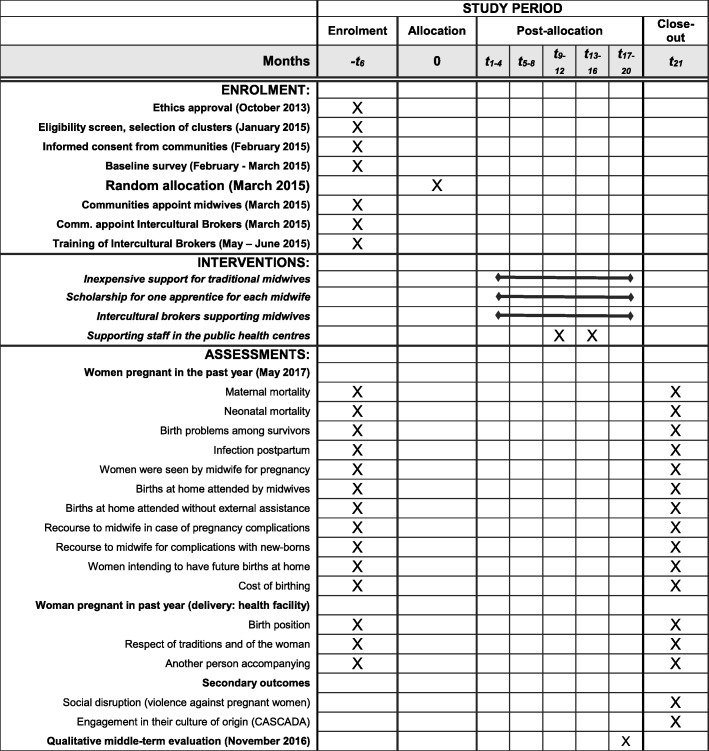
Schedule of enrolment, interventions and assessments for the study Safe Birth and Cultural Safety

The intervention will be coordinated by a local team based at CIET. The local team has more than 30 years of experience working in the rural areas of Guerrero. The intervention begins immediately after the training of intercultural brokers (component 4) and will continue for 2 years. Any change in the protocol will be notified to the registry of the trial (Fig. 2, Additional files 1 and 2).

Control communities receive usual healthcare services. Usual perinatal care for indigenous women in the Montaña region of Guerrero is provided by Western physicians (54.6%), nurses (4.2%) and traditional midwives without external support (20.7%); however, some 20.5% of these women do not have any antenatal care. Among those who received antenatal care, more than 3 out of ten women received less than five antenatal check-ups, the minimum indicated by Mexican standards [37].

In this region, Western physicians (36%), nurses (8%) and traditional midwives without external support (47.9%) provide usual care for childbirth. Some 8.1% of the indigenous women had other or no source of care [37]. Control municipalities have a healthcare centre (*hospital básico comunitario*) in San Luis Acatlán as well as two rural posts (*centro de salud rural* and *unidad de consulta externa*) in Atlixtac. In both cases, healthcare facilities are located in the population centres and provide services for the entire municipality. Women in remote areas need several hours’ walking or travel by gravel road to reach the closest healthcare facility. Regional general hospitals (Ometepec and Tlapa) attend the complicated cases remitted from these communities [38].

The intervention will become obvious to residents in the intervention sites, and some outcomes (particularly social cohesion) could be influenced by knowledge of intervention status. The main outcome indicators (non-inferiority for morbidity and maternal mortality) and other secondary outcomes would be less susceptible to this bias.

### Outcome measures

For objective (1), the central concern is the added benefit of supporting traditional midwives in a context of non-inferior maternal and neonatal mortality. The limited size of the populations involved hinders mortality estimates and increases reliance on intermediate outcomes: birth problems among survivors of pregnancy in the past year. We will measure maternal mortality and morbidity and neonatal mortality through direct questions in each household.

Secondary outcomes (objective (2)) include (a) reduction of social disruption, indicated by gender violence against pregnant women and (b) improvement in intermediate outcomes towards more engagement of women in their culture of origin. The CASCADA model describes these intermediate outcomes in a results chain based on the theory of planned behaviour, overcoming the well-documented limitations of the Knowledge, Attitude and Practices (KAP) model [39, 40]: *C*onscious knowledge, *A*ttitudes, positive deviation from *S*ubjective norms, intentions to *C*hange behaviour, *A*gency (individual and collective), *D*iscussion/socialisation of possible action and, finally, *A*ction or change of practice [41]. Two randomised trials in Pakistan and Mexico, a cross-sectional study in Southern Africa, and a qualitative analysis of narratives in three Southern African countries have used the CASCADA model [42–46].

In this case, the CASCADA model will reflect conscious knowledge of the traditional midwife, a positive attitude about using her services, a positive deviation from a negative subjective norm about traditional midwifery, intention to change in a future pregnancy, the agency to implement these choices, discussion of the choices with partners and, ultimately, interaction with the supported traditional midwife.

The economic outcome measures (objective (3)) are described below under ‘Economic analysis’.

The study will have two measurement points: a baseline survey administered by trained bilingual indigenous interviewers (February and March 2015) and a follow-up survey using the same procedure and questions about pregnancy experiences and outcomes to women pregnant during the past year (May 2017). The period of inquiry for the final survey is defined to avoid any overlap with the pre-intervention period. Given the extent of the region, logistical constraints mean it is not feasible to have continuous or mid-term data collection.

The surveys will use instruments tested during the pilot study and will include questions about: maternal deaths, neonatal deaths, number of times women are seen by the traditional midwife during pregnancy, proportion of births at home attended by midwives or without external assistance, frequency of recourse to the traditional midwife in case of pregnancy complications, frequency of recourse to the traditional midwife in case of complications with newborns, proportion of women intending to have future births at home, infection postpartum, and cost of birthing. Among women who gave birth in health institutions, we also will ask questions about their treatment, including birth position, availability of translators, presence of family members at the birth, presence of the traditional midwife at the birth, bathing in cold water, treatment of the placenta, retention of amulets, and how respectful they consider their treatment to have been.

Secondary outcomes measured in the follow-up survey will include: prevalence of violent acts towards pregnant women, opinion as to whom the woman should consult first when she learns that she is pregnant, opinion of who should attend to the woman first if she has complications during pregnancy, opinion as to who should decide whether to take the woman to the hospital if there are complications during childbirth, perception of neighbours’ preferences as to who should provide antenatal care, perception of neighbours’ preferences as to home vs institutional birth.

A qualitative mid-course peer evaluation using the Most Significant Change technique with local stakeholders will provide information about progress and the relevance of secondary outcomes regarding cultural safety [47]. This technique is a participatory method for monitoring and evaluation of complex projects in which participants narrate stories describing the most significant changes they attribute to the intervention, and implementers review the stories. This will provide information about change dynamics, identify issues in implementation and provide moral support for the intercultural brokers*.*

### Random allocation of the intervention

**Fig. 3:**
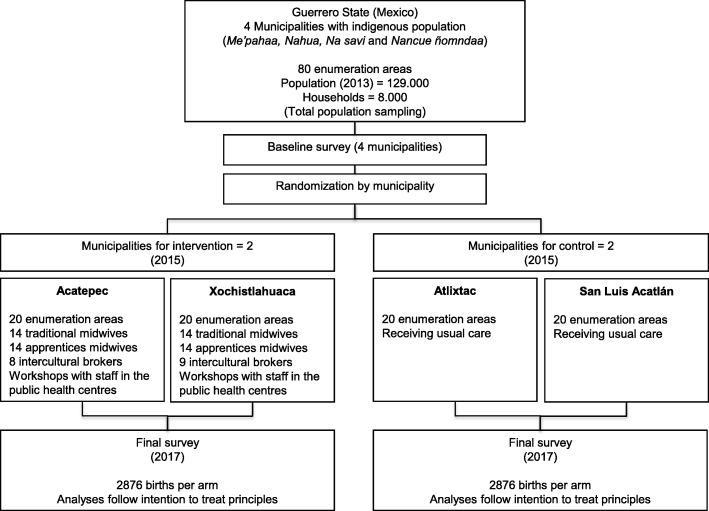
Flow diagram of the study Safe Birth and Cultural Safety

The total of 80 enumeration areas in the four municipalities are home to the four main indigenous groups (Fig. 3). If we allocated the intervention at the level of enumeration areas, we would expect a substantial contamination effect within each municipality (mothers from control enumeration areas going to authentic traditional midwives in the intervention enumeration areas) with strong spill-over influence within the same indigenous group served by the intervention midwives; through schools; and through local government or non-governmental organisations (NGOs) taking up the emerging evidence to guide interventions in control enumeration areas. This contamination would reduce the measured difference between control and intervention enumeration areas. To avoid this, the study will centrally randomise the intervention to two of the four municipalities (40 enumeration areas, 20 in each municipality).

### Analysis

#### Data entry and security

Independent operators will enter questionnaire responses twice, with verification of discordant entries from the original questionnaires. Researchers will check digitised data for logical errors. We will handle questionnaires from intervention and control sites in exactly the same way, with data technicians unaware of the intervention status of clusters.

#### Principal analysis

With 80 communities allocated evenly between the intervention and control arms, the principal analysis of primary outcomes will follow intention-to-treat principles using a cluster *t*-test (everyone included in each cluster, per allocation). We will report outcomes as absolute event rates among intervention and control groups, risk difference with two-sided 95% confidence intervals (95%CIs) and one-sided 97.5% confidence intervals for the non-inferiority analysis, and relative risk reduction (RRR) with 95%CI [48]. The intracluster correlation coefficient (ICC) will be calculated by dividing the between-cluster variance by the variance within and between clusters. 

*Sensitivity analysis* will focus on the different ethnic groups and their accompanying government health services. It will also examine the four intervention components separately because, although all components are available for all participating midwives, we expect a range of implementation in practice.

#### Secondary analysis

In each cluster, we will collect relevant data from the local government to determine rates of reported local crime and level of engagement in civic affairs. Individual-level data in a multilevel/hierarchical regression modelling technique will take into account group characteristics.

*Planned subgroup analysis and reporting* include a focus on the gender of the offspring. Age of the traditional midwife is also of interest because it is a core issue in the recovery of traditional healing and care practices.

The statistical analysis of data will rely on CIETmap, an open-source interface with the R programming language [49].

### Economic analysis

The economic dimension is not trivial. Intercultural dialogue can lead to new solutions for health promotion based on adequate use of local resources [50]. Evaluation of the work of traditional midwives should recognise that far fewer official resources support their work than support Western-trained birth attendants. Finally, cultural loss and depletion of natural resources around indigenous communities mean that some authentic traditional midwives cannot work at full capacity and in these cases we may need to implement some actions to strengthen traditional health systems or at least take into account this imbalance in the measurement process.

In 2 years, the intervention might change some population-based maternity outcomes, allowing aggregated costs to be compared between intervention and control municipalities. The concern is to quantify the somewhat increased cost of adding the intervention and the much-increased access this affords to indigenous women. A starting point is an assessment of site-specific maternal health services available to indigenous women from a societal perspective, based on (1) the implementation costs of these services and (2) the implementation costs of the Safe Birth and Cultural Safety project. Site visits and in-person interviews with representatives of services and of the project will assess local implementation costs. We will measure costs in Mexican pesos and convert into US$ to allow for international comparison.

From the results of the final survey, we will identify direct benefits in terms of maternal mortality and morbidity indicators, particularly birth complications. Additional benefits we expect to evaluate are (1) change in access/uptake of services and (2) secondary effects like increases in social capital, health literacy, or community planning skills in maternal health services. Finally, we will identify the completeness and timing of implementation to provide a context. We will express the relation between benefits and differences in costs using cost-effectiveness ratios [51].

A third component of the economic analysis will project the costs and effectiveness of implementing the project using alternative models of intervention to enhance sustainability. The specific objectives of this component will be to predict the most cost-effective strategy for wider implementation of Safe Birth and Cultural Safety. It will also help to identify the resources (including local government funding and community participation) needed for rollout.

### Missing data

All communities experience in-migration and out-migration. We will add new arrivals to the study but will not follow those leaving the clusters. We do not have reason to expect differential out-migration between intervention and control clusters. Self-selection (decision not to participate or not to answer certain questions) in the surveys is a concern. Those who opt not to respond may be less involved with safe motherhood initiatives – thus affecting the measured effect. Therefore, we will characterise subjects with missing data as far as possible and analyse the effect of missing data using the multiple imputation method in the Amelia II programme [52].

### Sample size calculation

Borrowing from the field of bioequivalence, equivalency trials and their statistical testing procedures focus on non-inferiority margins [53]. We expect that supporting traditional midwives does not result in worse primary outcomes of maternal health than does the available usual care in the region, principally because so few indigenous women in the study area access available services. Under the non-inferiority hypothesis, the trial might show equivalent or superior effects of the intervention [54]. The pilot study suggested additional benefits that secondary outcomes accrue from a culturally safe intervention. In the absence of previous studies in similar settings, we established a practical margin for non-inferiority-based discussion of findings with local authorities and indigenous communities. The resulting computation of study power illustrates the possibilities of demonstrating non-inferiority in these small communities of fixed size.

**Fig. 4:**
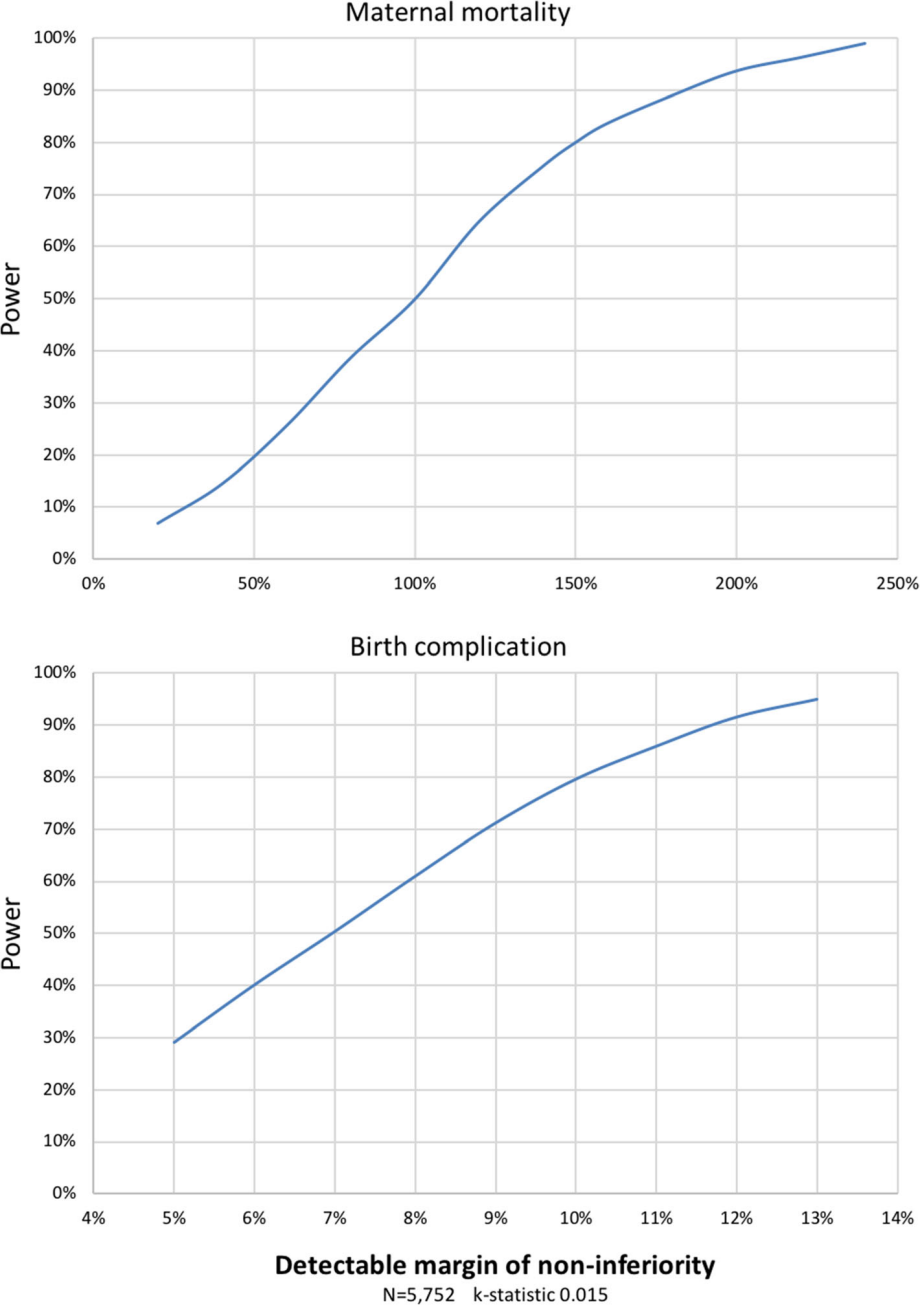
Power of the sample in relation to the margin of non-inferiority for maternal mortality and birth complications

Based on 2013 data, we expected 5752 births across the four municipalities [55]. This study size is too small to use maternal mortality as an outcome over the funded duration of the trial, using 150% as the minimum non-inferiority margin to be detected. For birth complications as primary outcome, this study size can detect differences within a practical margin for non-inferiority of 15%, with 80% power at a significance level of 5% (Fig. 4).

For the secondary outcome of improved skilled birth attendance (birth assisted by traditional midwife or physician), the pilot study suggested a rate of 92% in control communities (k-statistic 0.011). Follow-up of 1438 births in each cluster (two clusters per arm) would detect a 6.2% increase in skilled attendance (92.1% to 97.8%) with 80% power at a significance level of 5% (allowing us to set the non-inferiority margin at 15%).

These calculations assume no interaction effects with cluster as unit of primary analysis in unmatched parallel groups and relied on the trial simulator devised by Taylor and Bosch [56].

### Implications for health services

Strengthening traditional healthcare practices can lead to better maternal health outcomes for at least three reasons: a better use of local resources owned by communities; healthcare actions take into account the culture of the people and the environmental characteristics of the territory; and an increased level of cultural safety in obstetric care.

For many indigenous communities, place of birth and engagement in childbirth are deeply connected to their identity, viability of their cultures and territories, and their systems of governance [57, 58]. Some link the impositions associated with hospital births with marginalisation of their knowledge systems, and this can have serious social and cultural consequences [59, 60].

Traditional midwives hold detailed knowledge of each woman during her pregnancy, placing them in an ideal position to refer those who need specialised care like caesarean section to Western obstetricians [61, 62]. A system built on these synergies could result in less demand on already overloaded obstetric services, higher-quality care for those who need it and, with more resources available for those requiring surgical interventions, fewer post-delivery complications [16, 63].

### Ethical considerations

We do not anticipate adverse events or side effects. As communities in the pilot project adopted traditional midwives supported by the project, they continued to use government services for complications that traditional midwives do not deal with. In the pilot study significantly lower complication rates and infection rates among those using traditional midwives suggest improved referral and self-referral. There were no negative reactions from the government health services, which received increased referrals of high-risk cases.

The Ethics Committee of the *Centro de Investigación de Enfermedades Tropicales* of the *Universidad Autónoma de Guerrero* approved the trial on 22 October 2013 (Reference 2013–014). Community assemblies representing the indigenous peoples involved in the trial approved the project between January and February 2015. We adopted the ethical principles for medical research in indigenous communities proposed by the Research Group on Traditional Health Systems [64].

*Informed consent*: After clarification of the catchment area of each midwife, field coordinators from the project will identify a suitable community leader able to speak on behalf of the community. They will explain the proposed study and that the community might or might not be allocated to receive the intervention; then, they will seek their permission to include the community. This community leader will follow the traditional ways of the indigenous communities to reach the final decision about participation.

*Informed consent for interviews*: Trained interviewers will explain to respondents the nature of the study and the voluntary nature of their participation using suitable local language. They will explain that participants may decline to answer any questions that they do not wish to answer, may refuse to participate in the activity, and may end the interview at any time. Interviewers will clarify the procedures to ensure confidentiality. They will then ask respondents for oral informed consent for the interview. The informed consent is oral because of the high rates of illiteracy among the participants.

*There will be no biological samples taken*.

*Ensuring confidentiality*: Fieldworker and data operator training will emphasise their responsibility for maintaining confidentiality of all information accessed during the work. We will report grouped findings in a way that does not allow identification of any individuals or communities. No names or identifiers will be recorded next to individual questionnaire responses and reports of findings will not identify individual communities.

*Protection of emotional well-being*: It is possible that questions about infant and maternal deaths could awaken distressing memories. If this happens, the interviewer will stop the interview, assess the condition of the respondent, use words of support, and immediately inform the field coordinator. The field coordinator will inform the project coordinator (an experienced researcher and physician) to decide the actions needed to ensure the welfare of the participant. The field coordinator will be responsible for ensuring that these actions are completed. Our experience suggests that the opportunity to engage in household and community protection to be uplifting and a self-affirmation for participants. We will provide specific training for interviewers about asking sensitive questions.

*Normative pressure within communities*: The pilot revealed no pressure on women to seek help from the traditional midwives in intervention communities. However, government conditional cash transfer programmes may have a strong influence towards choosing to use government supported health centres. The clinical practice of the staff in the government health centres in the intervention municipalities will remain independent of the project.

*Data security*: Digital records will be secure and accessible only to the principal investigators. Original paper records will be securely transported, stored, retained and finally destroyed in accordance with CIET guidelines for security, storage and eventual destruction of paper records.

## Discussion

Recent studies in indigenous communities confirm the importance of sociocultural dimensions of safe motherhood [57, 60, 65, 66]. Most indigenous communities face a transition from ancient traditions to Western culture, implying dual healing resources and a complex process of health choices [67]. This cultural transition involves changes in education and service delivery but is an incomplete process in many places, leaving important gaps [68]. For example, indigenous people shun Western services as a reaction to feeling that their culture and values are ignored [20, 57, 59, 69]. Women in these settings fall between the two cultures, where traditional services are attenuated if not actively undermined, but where there is not full access to Western services [70]. Therefore, promotion of intercultural dialogue could open a way for indigenous women to think more highly of Western services and to use them more efficiently [68].

The support requested by the traditional midwives during co-design of the intervention in the pilot study included an apprenticeship programme. In this important sense, authentic traditional midwives represent renewal of their communities and the intergenerational transfer of traditional midwifery skills. Traditional midwives might be a factor in social cohesion, in marital harmony or in the socialisation of young people. Support for traditional midwives means support of recovery and reinforcement of authoritative indigenous knowledge.

Most recent research follows the convention of ‘upgrading’ the skills of traditional midwives in Western concepts of safe motherhood [63, 71–73]. Often, these approaches rely on ill-suited methods and often inappropriate teachers – a young Western nurse who is supposed to ‘teach’ women three times her age – and who might dismiss or discourage indigenous practices [20, 61]. This limited understanding of tradition and culture has had deleterious effects on traditional midwifery roles [19, 72]. This trial shifts the focus to support for, rather than replacement or reinvention of traditional midwives. We are not aware of accounts of other trials taking this approach.

Training local community leaders as intercultural brokers (*técnicos interculturales*) to bridge the intercultural gap is the centrepiece of the trial. Our approach is to foster intercultural dialogue in support of both the traditional midwife and the Western obstetrician, each to do what they do best. The argument has never been that traditional midwives might carry out caesarean sections, nor that Western obstetricians are well placed to support indigenous women on issues like work in pregnancy or intimate partner violence. It makes sense to combine primary, secondary and tertiary prevention of maternal morbidity and mortality through an adequate interaction between the two health systems.

Community health workers have long been recognised as ‘relevant to most service delivery priorities at the primary healthcare level, particularly in under-served areas’ [74]. The intervention does not seek to train community workers to deliver clinical services, but rather to train intercultural brokers to liaise between communities and health services, especially for promoting prevention strategies for maternal and child morbidity [75]. This will be the first trial providing information about the value of this sort of training of intercultural brokers in improving maternal outcomes.

This trial might contribute to the discipline of intercultural epidemiology by adapting high-value epidemiological methods to study traditional medical practices in remote indigenous settings. Safe motherhood in cultural safety must go beyond simply classifying indigenous women as high risk, and beyond the degrading concept of ‘otherness’ implicit in cultural sensitivity and cultural competence [76]. A culturally safe approach recognises traditional culture as an asset and the damaging effect that cultural loss and disempowerment have on health status of individuals and communities [77]. Although traditional health systems remain in widespread use [78], evidence about their health impact is scarce and we need attuned epidemiological methods to understand them [79]. We plan to disseminate our results in academic settings as well as to communicate evidence to communities through the intercultural brokers.

Advances of this protocol include use of the pragmatic RCT design, with large clusters (entire municipalities) reducing the contamination of control communities. The involvement of traditional midwives in designing the intervention is likely to be crucial to its success. This is an example of developing better practices of intercultural health based on a respectful intercultural dialogue [35, 80].

### Trial status

Research protocol, 28 February 2017.

Recruitment start date: 1 July 2015; recruitment end date: 31 May 2017.

## Additional files

Additional file 1: Standard Protocol Items: Recommendations for Interventional Trials (SPIRIT) 2013 Checklist: recommended items to address in a clinical trial protocol and related documents: Safe Birth and Cultural Safety. Description of data: SPIRIT 2013 Checklist completed. (DOC 123 kb)

Additional file 2: WHO Trial Registration Data Set (Version 1.2.1): Safe Birth and Cultural Safety. Description of data: information about the study regarding WHO Trial Registration Data Set. (DOC 43 kb)

## Abbreviations

CASCADA: *Conocimientos Actitudes normas Subjetivas intención de Cambiar Agencia Discusión Acción* (Conscious knowledge Attitudes Subjective norms intention to Change Agency Discussion Action)
CIET: *Centro de Investigación de Enfermedades Tropicales* (Tropical Disease Research Centre) at the Universidad Autónoma de Guerrero, in Mexico
ICC: Intracluster Correlation Coefficient
KAP: Knowledge, Attitude and Practices
NNT: Number Needed to Treat
RCT: Randomised controlled trial
RRR: Relative risk reduction
TBA: Traditional birth attendant
