# Supplementary material for: Safe Birth and Cultural Safety in southern Mexico: study protocol for a randomised controlled trial
Source: Trials. 2018 Jul 4;19:354. doi: 10.1186/s13063-018-2712-6 (PMC6033215; doi:10.1186/s13063-018-2712-6)
Supplement: Supplementary file 2 — WHO Trial Registration Data Set (Version 1.2.1): Safe Birth and Cultural Safety. Description of data: information about the study regarding WHO Trial Registration Data Set. (DOC 43 kb) [file 13063_2018_2712_MOESM2_ESM.doc]

Additional file 2

WHO Trial Registration Data Set (Version 1.2.1): Safe birth in cultural safety

| Primary Registry and Trial Identifying Number | ISRCTN 12397283 |
| --- | --- |
| Date of Registration in Primary Registry | 06/12/2016 |
| Secondary Identifying Numbers | BMx2 |
| Source(s) of Monetary or Material Support | The National Council of Science and Technology of Mexico (CONACyT) |
| Primary Sponsor | Tropical Disease Research Centre, University of Guerrero  Calle Pino s/n, Colonia El Roble, código postal 39640 (Acapulco, Mexico) |
| Secondary Sponsor(s) | CIET-PRAM, Department of Family Medicine, McGill  Centre for Intercultural Medical Studies |
| Contact for Public Queries | Ivan Sarmiento  Ivan.sarmiento@mail.mcgill.ca  +1 (438) 9278710  5858 Chemin de la Côte-des-Neiges 3rd Floor, Suite 300, H3S 1Z1 (Montreal, Canada) |
| Contact for Scientific Queries | Neil Andersson  neil.andersson@mcgill.ca  +1 (438) 9278710  5858 Chemin de la Côte-des-Neiges 3rd Floor, Suite 300, H3S 1Z1 (Montreal, Canada) |
| Public Title | Safe birth in cultural safety |
| Scientific Title | Safe motherhood in cultural safety – intercultural dialogue in support of indigenous midwifery in southern Mexico: pragmatic cluster-randomised controlled trial (BMx2) |
| Countries of Recruitment | Mexico |
| Health Condition(s) or Problem(s) Studied | Maternal and neonatal mortality, pregnancy and birth problems |
| Intervention(s) | 1. Material support for authentic traditional midwives to strength their practice and foster the intergenerational transfer of their profession  2. Scholarship support of one apprentice for each midwife  3. Supporting staff in the public health centres of each of the intervention municipalities to improve understanding and attitudes toward authentic traditional midwives  4. Training of intercultural brokers |
| Key Inclusion and Exclusion Criteria | All women in the baseline sample clusters who give birth or become pregnant during the trial period  All adult family members of the above |
| Study Type | Cluster Randomized Trial |
| Date of First Enrollment | 1st July 2015 |
| Target Sample Size | 80 enumeration areas with about 8000 households |
| Recruitment Status | Recruiting: participants are currently being recruited and enrolled |
| Primary Outcome(s) | 1. Among women pregnant in the past year  Maternal morbidity  Maternal mortality  Neonatal mortality  Birth problems among survivors  Infection postpartum  Social disruption (gender violence)  Number of times women were seen by midwife during pregnancy  Births at home attended by midwives  Births at home attended without external assistance  Recourse to midwife in case of pregnancy complications  Recourse to midwife in case of complications with new-borns  Women intending to have future births at home  Cost of birthing  2. Among women pregnant in past (delivery in health facility)  Birth position  Availability of translators  Presence of family members at birth  Presence of midwife at birth  Bathing in cold water  Treatment of the placenta  Retention of amulets  How respectful they considered their treatment to have been |
| Key Secondary Outcomes | Secondary outcomes  Violent acts toward pregnant women  Opinion as to whom the woman should consult first when she learns she is pregnant  Opinion as to who should attend the woman first if she has complications during pregnancy  Opinion as to who should decide whether to take the woman to the hospital if there are complications during childbirth  Perception of neighbours' preferences as to who should provide antenatal care  Perception of neighbours' preferences as to home vs institutional birth |
